# Supplementary material for: High-resolution structures of malaria parasite actomyosin and actin filaments
Source: PLoS Pathog. 2022 Apr 4;18(4):e1010408. doi: 10.1371/journal.ppat.1010408 (PMC9037914; doi:10.1371/journal.ppat.1010408)
Supplement: S1 Fig — (A) Representative micrographs of MyoA-decorated Act1 filaments. (B) Reference free classes derived from the micrographs. (C) Fourier shell correlation of the Act1:MyoA complex. Using the 0.143 Fourier shell threshold criterion, the global resolution is 3.1 Å. The masked curve was calculated from independently refined half-datasets with a soft-mask filtered to 15 Å. (PDF) [file ppat.1010408.s001.pdf]

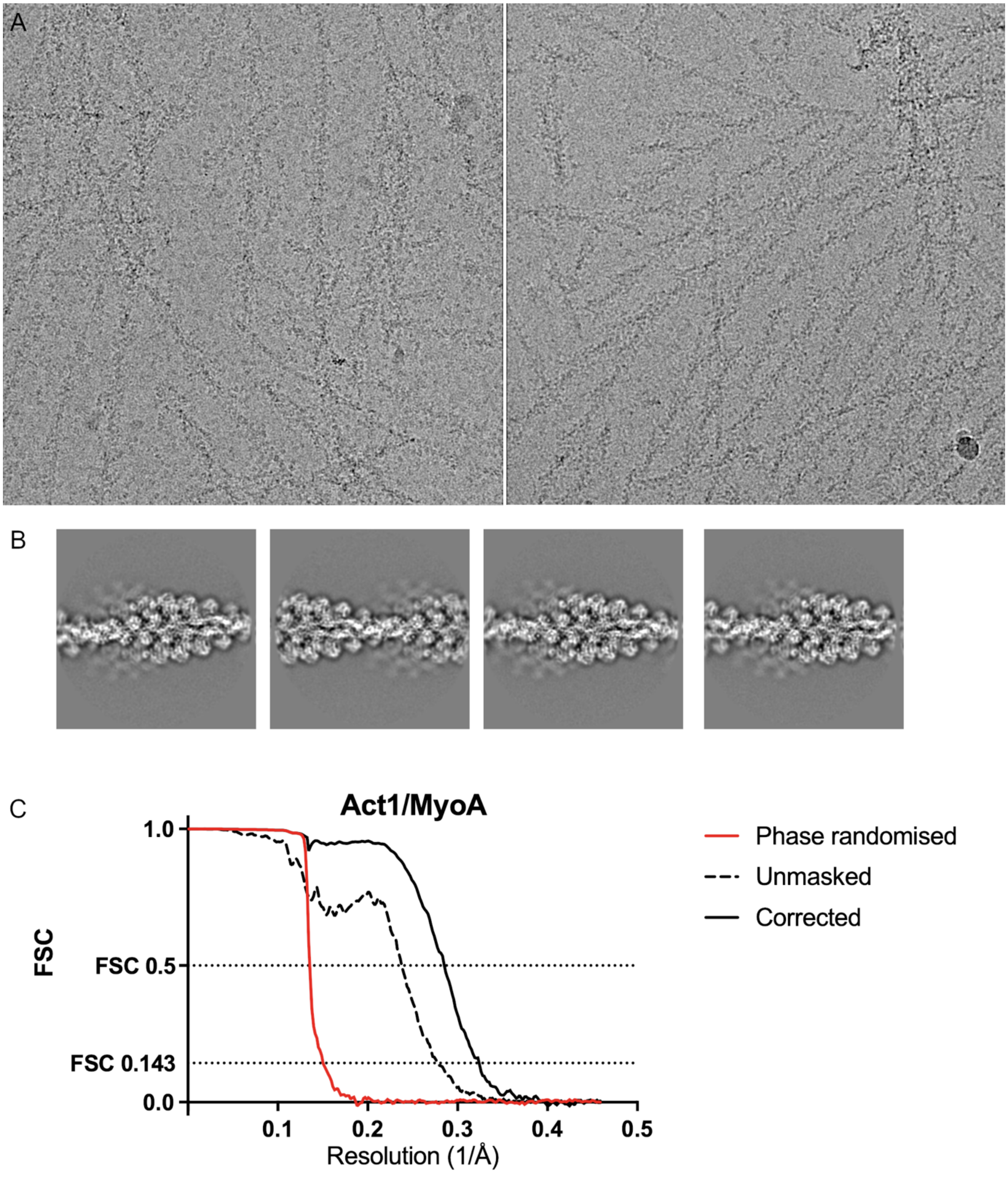

S1 Fig. Act1:MyoA filaments and resolution reconstruction. (A) Representative micrographs of MyoA-decorated Act1 filaments. (B) Reference free classes derived from the micrographs. (C) Fourier shell correlation of the Act1:MyoA complex. Using the 0.143

Fourier shell threshold criterion, the global resolution is 3.1 Å. The masked curve was calculated from independently refined half-datasets with a soft-mask filtered to 15 Å.
