# Supplementary material for: High-resolution structures of malaria parasite actomyosin and actin filaments
Source: PLoS Pathog. 2022 Apr 4;18(4):e1010408. doi: 10.1371/journal.ppat.1010408 (PMC9037914; doi:10.1371/journal.ppat.1010408)
Supplement: S2 Fig — (A) Representative micrographs of Act1 filaments low-pass filtered at absolute frequency 0.15, (B) reference free classes derived from them, and (C) Fourier shell correlation of the Act1 filament. Using the 0.143 Fourier shell threshold criterion, the global resolution is 2.6 Å. The corrected curve was calculated from independently refined half-datasets with a soft-mask filtered to 15 Å in Relion. (PDF) [file ppat.1010408.s002.pdf]

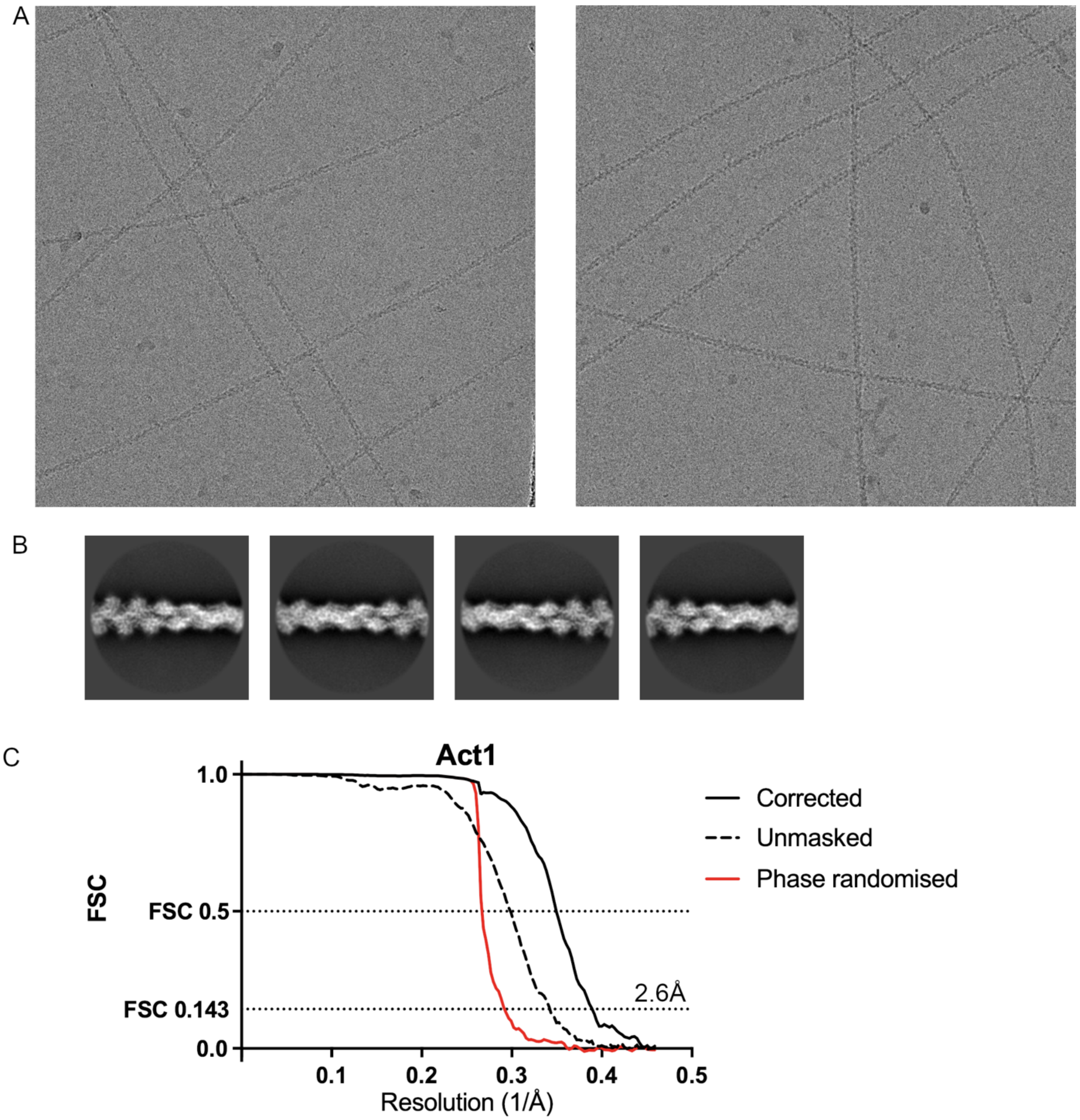

S2 Fig. Examples of the Act1 filaments and resolution reconstruction. (A) Representative micrographs of Act1 filaments low-pass filtered at absolute frequency 0.15, (B) reference free classes derived from them, and (C) Fourier shell correlation of the Act1 filament. Using the 0.143 Fourier shell threshold criterion, the global resolution

is 2.6 Å. The corrected curve was calculated from independently refined half-datasets with a soft-mask filtered to 15 Å in Relion.
