# Supplementary material for: High-resolution structures of malaria parasite actomyosin and actin filaments
Source: PLoS Pathog. 2022 Apr 4;18(4):e1010408. doi: 10.1371/journal.ppat.1010408 (PMC9037914; doi:10.1371/journal.ppat.1010408)
Supplement: S3 Fig — The local resolution estimation is based on the Fourier shell correlation threshold 0.3 calculated with Blocres in the Bsoft software package, which was displayed on the final sharpened map. (PDF) [file ppat.1010408.s003.pdf]

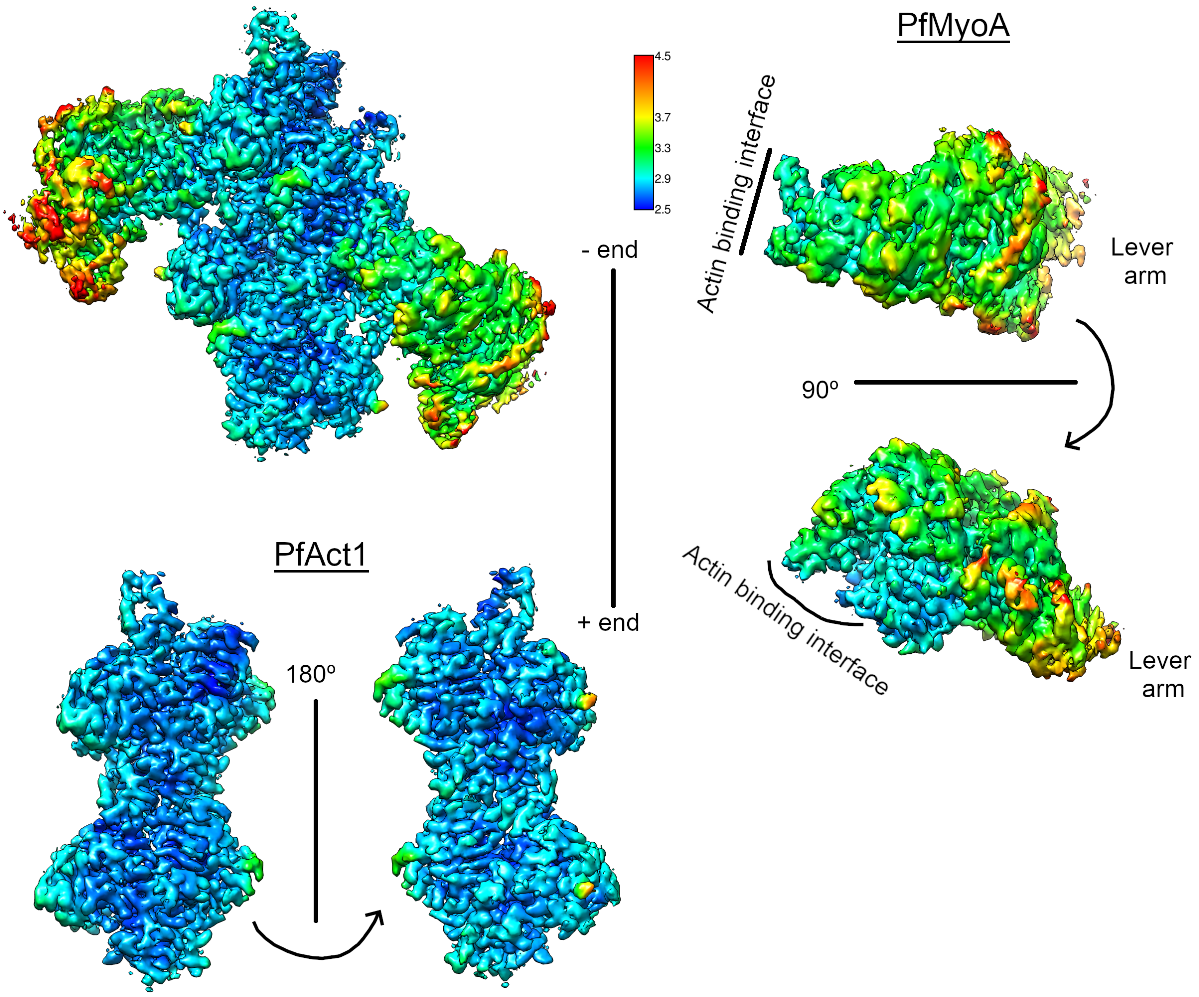

S3 Fig. Local resolution of the MyoA-decorated Act1 filament. The local resolution estimation is based on the Fourier shell correlation threshold 0.3 calculated with Blocres in the Bsoft software package, which was displayed on the final sharpened map.
