# Supplementary material for: High-resolution structures of malaria parasite actomyosin and actin filaments
Source: PLoS Pathog. 2022 Apr 4;18(4):e1010408. doi: 10.1371/journal.ppat.1010408 (PMC9037914; doi:10.1371/journal.ppat.1010408)
Supplement: S4 Fig — The local resolution estimation is based on Fourier shell correlation threshold 0.3 calculated with Blocres in the Bsoft software package, displayed on the final sharpened map. The left panel shows a central section of the filament, and the right panel shows an individual actin protomer with the ligand densities highlighted. (PDF) [file ppat.1010408.s004.pdf]

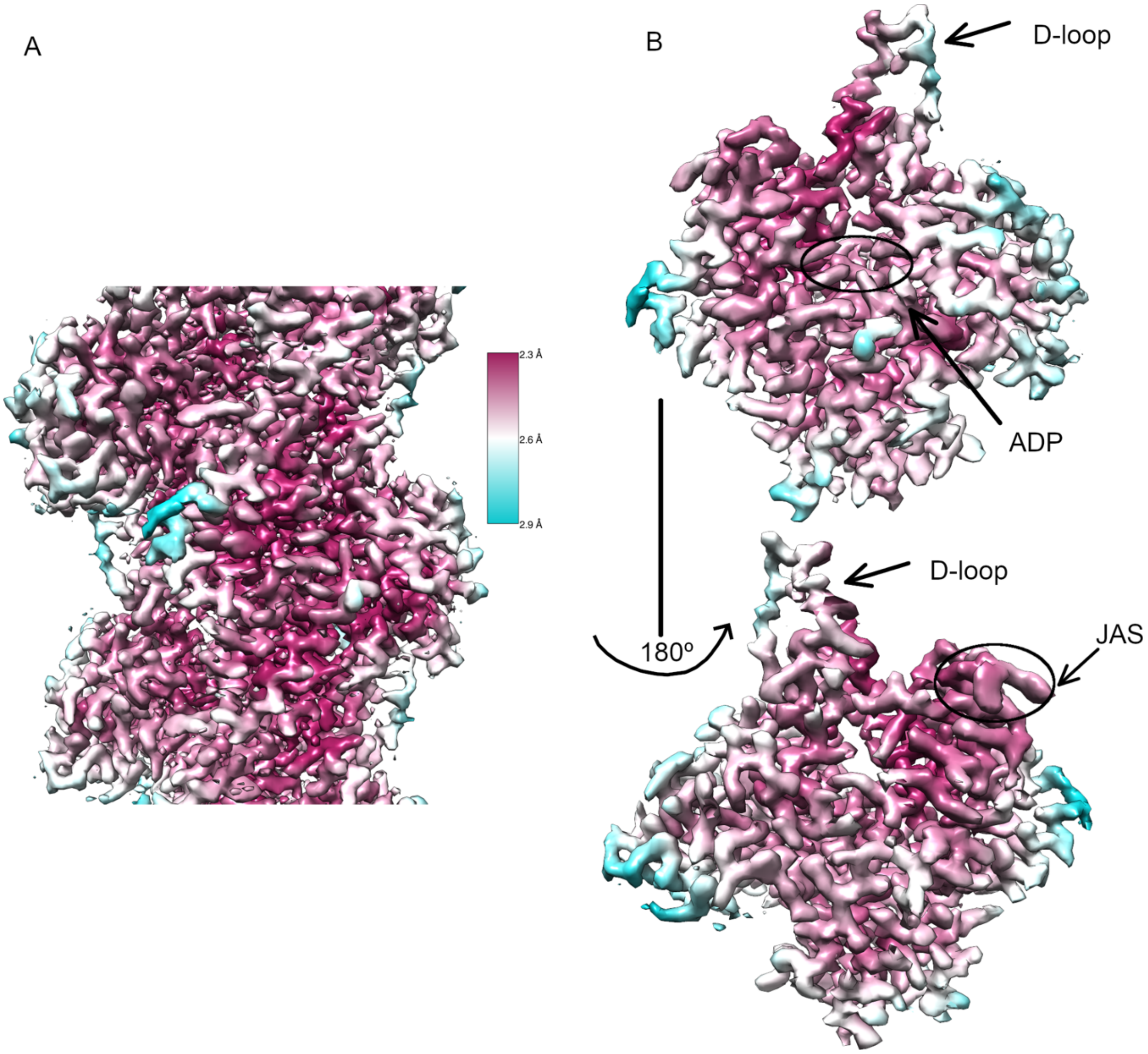

S4 Fig. Local resolution of the Act1 filament. The local resolution estimation is based on Fourier shell correlation threshold 0.3 calculated with Blocres in the Bsoft software package, displayed on the final sharpened map. The left panel shows a central section of the filament, and the right panel shows an individual actin protomer with the ligand densities highlighted.
