# Supplementary material for: High-resolution structures of malaria parasite actomyosin and actin filaments
Source: PLoS Pathog. 2022 Apr 4;18(4):e1010408. doi: 10.1371/journal.ppat.1010408 (PMC9037914; doi:10.1371/journal.ppat.1010408)
Supplement: S5 Fig — (PDF) [file ppat.1010408.s005.pdf]

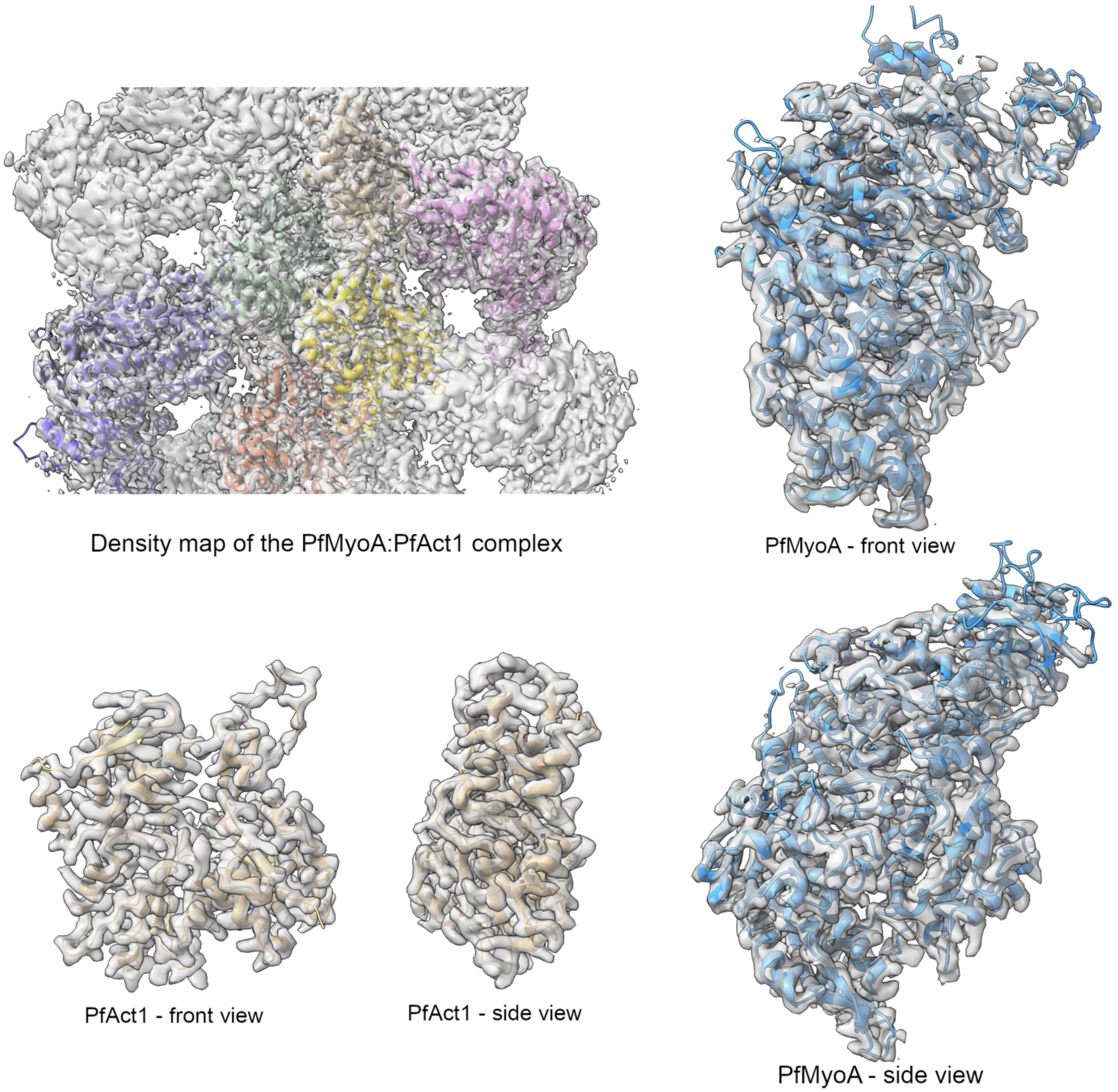

S5 Fig. Density maps of the Act1:MyoA complex and the individual sub-units.
