# Supplementary material for: High-resolution structures of malaria parasite actomyosin and actin filaments
Source: PLoS Pathog. 2022 Apr 4;18(4):e1010408. doi: 10.1371/journal.ppat.1010408 (PMC9037914; doi:10.1371/journal.ppat.1010408)
Supplement: S6 Fig — (Upper panel) The MyoA motor domain in two slightly different orientations to display the different functional motifs, shown in different colors and labeled. (Lower panel) In the structure-based sequence alignment, strictly conserved residues have red background, more than 70% identical residues are red in a blue frame. Amino acids in the actomyosin interface are indicated with cyan shading. Above the sequence, the actin-contacting structural motifs are indicated. The black bars under the sequence show fractional buried surface area for MyoA residues (1 bar <30%, 2 bars 30%-60%, 3 bars >60%). The sequence alignment is based on pairwise structural alignment against MyoA using the DALI server [Holm L. DALI and the persistence of protein shape. Protein Sci. 2020;29(1):128–40. Epub 2019/10/14. doi: 10.1002/pro.3749] [54], and further annotated using the ESPript 3.0 web interface [Robert X, Gouet P. Deciphering key features in protein structures with the new ENDscript server. Nucleic Acids Res. 2014;42(Web Server issue):W320-4] [75]. (PDF) [file ppat.1010408.s006.pdf]

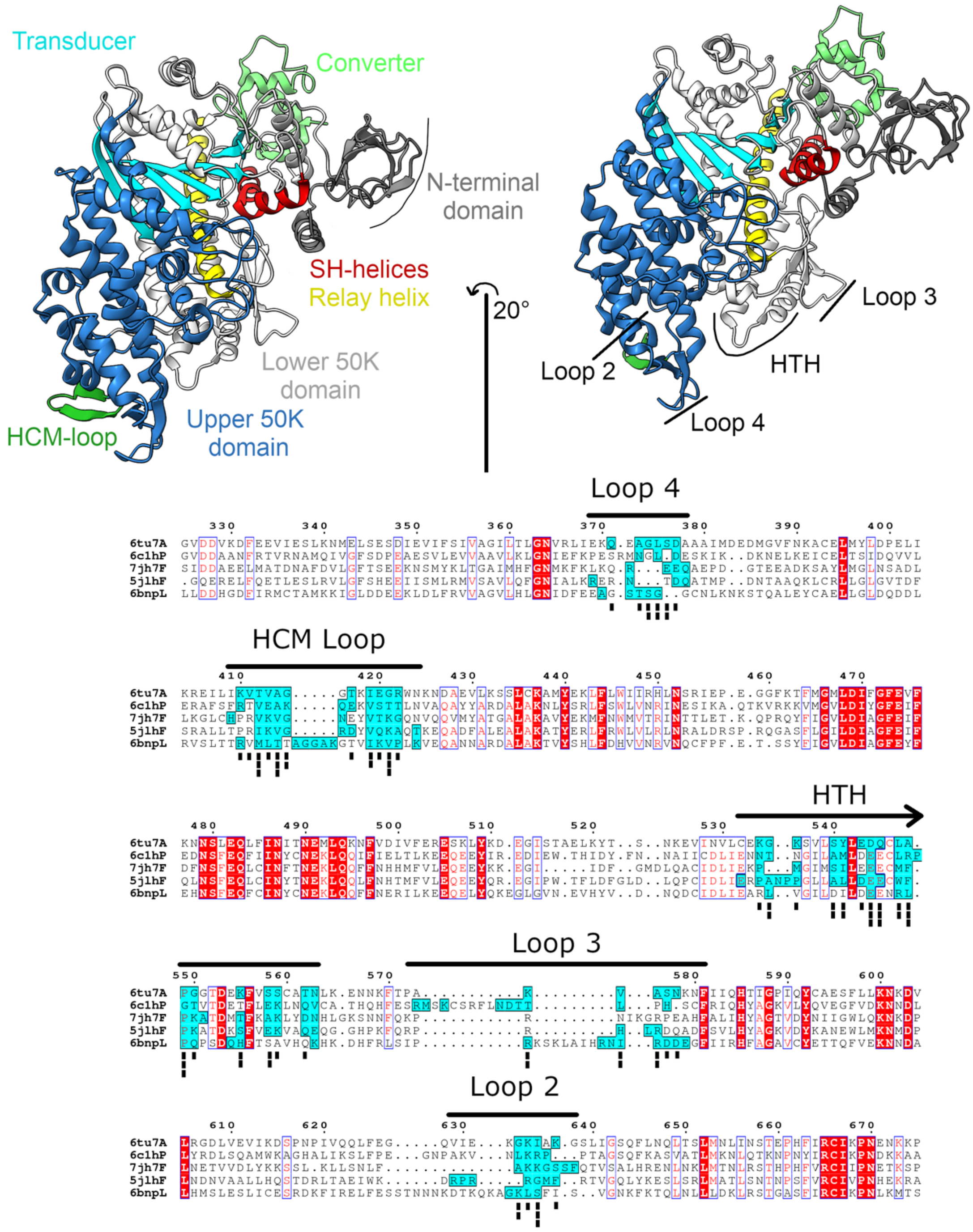

S6 Fig. Structural domains and sequence comparison of the MyoA motor domain. (Upper panel) The MyoA motor domain in two slightly different orientations to display the different functional motifs, shown in different colors and labeled. (Lower panel) In the structure-based sequence alignment, strictly conserved residues have red background,

more than 70% identical residues are red in a blue frame. Amino acids in the actomyosin interface are indicated with cyan shading. Above the sequence, the actin-contacting structural motifs are indicated. The black bars under the sequence show fractional buried surface area for MyoA residues (1 bar <30%, 2 bars 30%-60%, 3 bars >60%). The sequence alignment is based on pairwise structural alignment against MyoA using the DALI server [Holm L. DALI and the persistence of protein shape. Protein Sci. 2020;29(1):128-40. Epub 2019/10/14. doi: 10.1002/pro.3749], and further annotated using the ESPript 3.0 web interface [Robert X, Gouet P. Deciphering key features in protein structures with the new ENDscript server. Nucleic Acids Res. 2014;42(Web Server issue):W320-4].
