# Supplementary material for: High-resolution structures of malaria parasite actomyosin and actin filaments
Source: PLoS Pathog. 2022 Apr 4;18(4):e1010408. doi: 10.1371/journal.ppat.1010408 (PMC9037914; doi:10.1371/journal.ppat.1010408)
Supplement: S7 Fig — Actomyosin structures were analyzed using PDBePISA [Krissinel E, Henrick K. Inference of macromolecular assemblies from crystalline state. J Mol Biol. 2007;372(3):774–97] [55]. A structure-based sequence alignment for each myosin is shown in the vertical column. The fractional buried surface area is shown for each amino acid residue in the actin interface. Long horizontal red boxes show common interfaces (fractional BSA >25% in all six structures). Grey boxes highlight additional interactions, which are discussed in the main text. Green and magenta boxes around amino acid residues indicate conserved and ancillary, respectively, interfaces, as defined by Robert-Paganin et al. [23]. (PDF) [file ppat.1010408.s007.pdf]

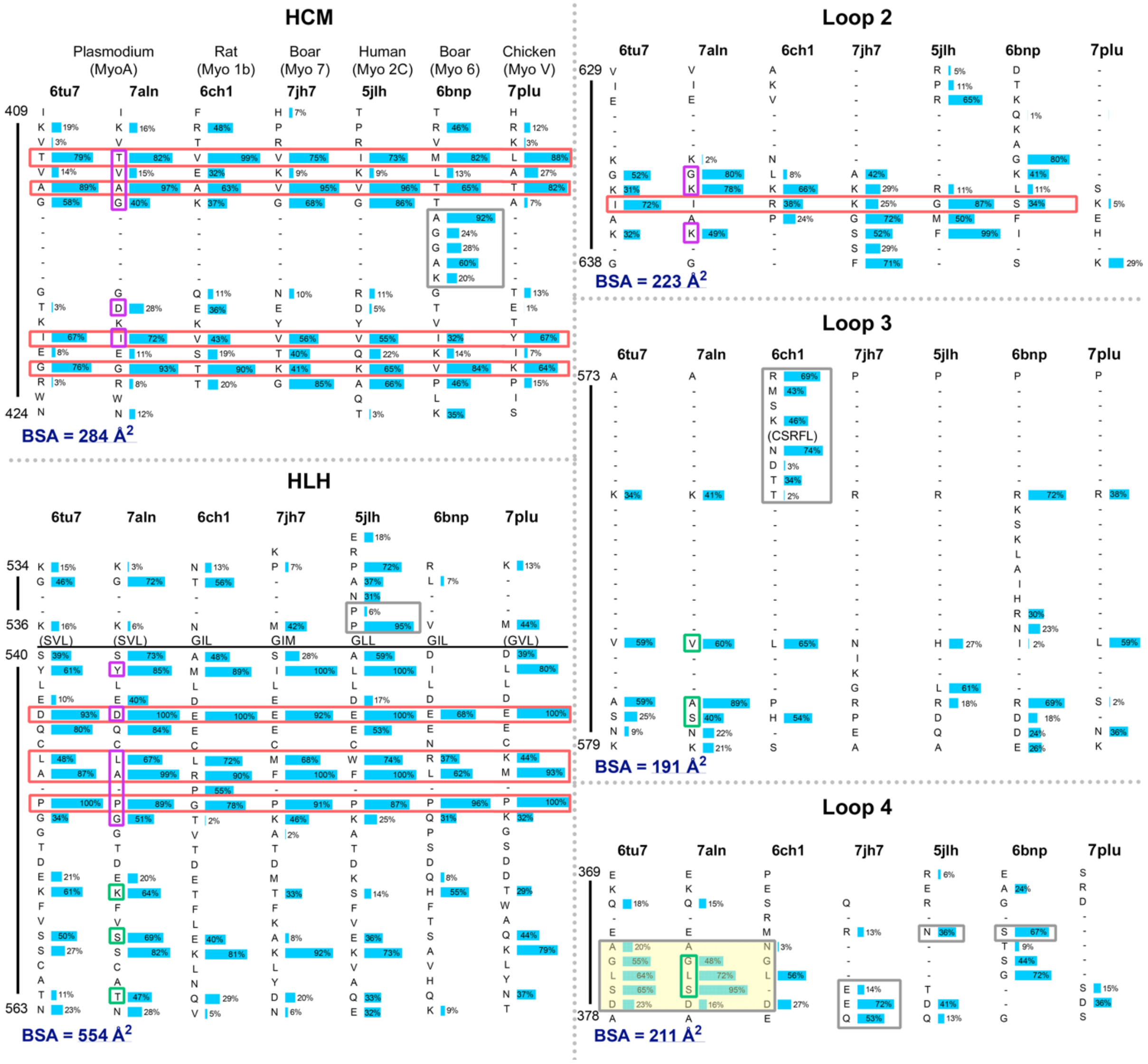

S7 Fig. Buried surface areas of different actomyosin complexes in the rigor conformation. Actomyosin structures were analyzed using PDBePISA [Krissinel E, Henrick K. Inference of macromolecular assemblies from crystalline state. J Mol Biol. 2007;372(3):774-97]. A structure-based sequence alignment for each myosin is shown in the vertical column. The fractional buried surface area is shown for each amino acid residue in the actin interface. Long horizontal red boxes show common interfaces (fractional BSA >25% in all six structures). Grey boxes highlight additional interactions, which are discussed in the main text. Green and magenta boxes around amino acid

residues indicate conserved and ancillary, respectively, interfaces, as defined by Robert-Paganin *et* al. [Robert-Paganin J, Xu XP, Swift MF, Auguin D, Robblee JP, Lu H, et al. The actomyosin interface contains an evolutionary conserved core and an ancillary interface involved in specificity. Nat Commun. 2021;12(1):1892].
