# Supplementary material for: High-resolution structures of malaria parasite actomyosin and actin filaments
Source: PLoS Pathog. 2022 Apr 4;18(4):e1010408. doi: 10.1371/journal.ppat.1010408 (PMC9037914; doi:10.1371/journal.ppat.1010408)
Supplement: Attachment — Submitted filename: rebuttal2.pdf [file ppat.1010408.s014.pdf]

Dear Dr. Soldati-Favre,

please find below our responses to the reviewer comments.

Sincerely,

Inari Kursula and Peter Rosenthal, on behalf of all authors

—

Reviewer # 2 recommends a revision of the glideosome model and a discussion on the possible rearrangements necessary to fit within the PM-IMC space while reviewer # 3 recommends some trimming

R: We thank the reviewers and editors for the positive comments on the revised version of our manuscript. We have addressed the mentioned reviewer comments as detailed below.

Reviewer #1: Paper has been improved and supplemented with new data

Reviewer #2: The revised manuscript from Vahokoski addresses most of the comments and suggestions made by the reviewer. The authors have compared in more details their structure with the published structure from Robert-Paganin et al. (pdbid: 7aln) and added an in vitro experiment measuring the power stroke by optical trapping, showing that the actomyosin light chain complex produces a power stroke similar to other myosins with two light chains. They also modified figures to include additional data and provide more accuracy on certain points.

They have placed GAC within the PM-IMC space, giving an idea of how much space this protein would occupy when connecting actin filaments with the PM. Unfortunately, the illustration presented in figure 2B, with GAC being distant from both the PM and actin, suggests that placement of GAC in incompatible with the model, or that the structure of GAC is not accurate. The text mentions this issue (line 194) but publishing such a model can be confusing for readers "In the parasite, when the additional space taken up by the actin filament (diameter ~6.5 nm) and GAC (Dmax ~25 nm; [16]) is considered, it is clear that there must be significant geometric and steric constraints on how the actomyosin complex might be arranged".

R: We agreed with reviewer suggestions to include GAC in figure 2, and this appears to have satisfied referees 1 and 3. To make our point clearer, in line with referee 2's additional concern, we have now further revised the figure to make GAC more schematic to solely indicate its dimensions relative to the PM-IMC space. We have also indicated clearly in the legend that the schematic is based on the SAXS envelope calculated in cited reference (16): "An outline of GAC (dotted lines) in two randomly chosen orientations indicates approximate dimensions derived from the *T. gondii* GAC small-angle X-ray scattering (SAXS) low-resolution envelope [16]"**.**

The main text also now says: "Also the structure of GAC may be different in the

tightly packed pellicle than in solution"**.**

Authors have identified a cavity within the F-actin structure that potentially could be occupied by non-identified solute. In the revised manuscript they discuss disease-associated mutations lining the cavity, supporting the importance of the pocket for F-actin function. Unfortunately, no in vitro or in vivo assays with mutated actins were performs to support their hypotheses.

R: These additional experiments are very time-consuming and best addressed in a separate study.

Phosphorylation status of Ser19 has been addressed by MS, showing a 50% of residues carrying this PTM.

Absence of phosphorylation of Thr417 in the current structure, differing from the 7aln structure where a phosphomimetic Thr417Asp mutation was necessary, is better addressed in the current manuscript

Reviewer #3: The authors have addressed all my previous comments.
The authors essentially addressed all my previous suggestions. All fine from that side. To my eyes, an unfortunate development was that they added an additional experiments, meant to address reviewers 1 and 2, which does not seem to answer any question in the manuscript. They do not exploit those results for much either, so if feels a little pointless. I think the paper would seriously benefit from some trimming.

R: The new optical trapping data, in addition to providing new insight into parasite actomyosin function, also demonstrates that the complex we have used for structure determination is functional, which we feel strengthens the manuscript. We have now stated this more clearly in the revised manuscript as follows: "To confirm that our recombinant MyoA:ELC:MTIP complex was fully-functional, we measured its ATP-driven power stroke, using single-molecule optical trapping nanometry [42] (see Materials and Methods)"**. As the reviewer suggests, we have significantly trimmed the main text part of this discussion because much of the technical details on the experiment are clear in the methods section.**
