# Supplementary material for: High-resolution structures of malaria parasite actomyosin and actin filaments
Source: PLoS Pathog. 2022 Apr 4;18(4):e1010408. doi: 10.1371/journal.ppat.1010408 (PMC9037914; doi:10.1371/journal.ppat.1010408)
Supplement: Attachment — Submitted filename: response_to_reviewers.docx [file ppat.1010408.s013.docx]

Dear Dr. Soldati-Favre,

please find below our responses to the reviewer comments.

Sincerely,

Inari Kursula and Peter Rosenthal, on behalf of all authors

Reviewer's Responses to Questions

**We thank the editors and reviewers for the positive comments on our manuscript and suggestions for its improvement. We have described changes in our point-by-point response below. In summary, we have provided new analyses as requested, made corrections, measurements on the power stroke of MyoA complexed with light chains, and confirmed the phosphorylation state of Ser19.**

**Part I - Summary**
Please use this section to discuss strengths/weaknesses of study, novelty/significance, general execution and scholarship.

Reviewer #1: The focus of this paper is the use of electron microscopy in the structure determination of the actomyosin motor complex from Plasmodium falciparum. The complex includes the parasite actin filament decorated with the class XIV myosin motor and two associated light-chains.

The structures provide genuine new insight into variety of aspects: i) the myosin-actin interface in atomic detail, ii) the light-chain binding region, including the essential light chain and the myosin tail interacting protein and iii) the arrangement of the actomyosin motor complex in the sub-pellicular compartment.

Slightly disappointing that there were no biological experiments to probe the structural observations at the myosin-actin and light chain interfaces. If any updates on this are available or feasible within a reasonable timeframe it would enhance the paper. Despite this, and although this paper is a pure structure biology study, it is a significant step forward in our understanding of the actomyosin complex and is therefore suitable for publication in Plos Path.

**R: We thank the reviewer for appreciating our work and supporting publication. We now include additional functional experiments in the form of optical trapping assays.**

Reviewer #2: The manuscript from Vahokoski et al. describes the 3.1 angstroem resolution structure of plasmodium actin filament decoreated with MyoA motors. PfMyoA is essential for parasite entry into red blood cells and as such represent an appealing drug candidate. The manuscript also describes the higher resolution of a bare plasmodium actin filament. The manuscript is well written with detailed atomic description of the structures. The data nicely show that plasmodium MyoA-actin interaction shares highly similar structure with other actomyosin structures, although there is large divergence between plasmodium and mammalian myosin motor sequences. Additionally, the structure of the myosin-actin complex is almost superimposable with the isolated structures of either PfAct1 filaments or of the MyoA protein. The high resolution structure of plasmodium actin filament identified a unique small cavity absent in other actin structures that could accommodate a non-identified solute.

Overall the results are highly similar to what was published in March 2021 (Robert-Paganin J. et al. Nat. Commun) and do not bring much additional information. The current manuscript emphasizes that the two myosin light chains ELC1 and MLC1 are present in their CryoEM reconstruction, but the very low resolution of these proteins does not add details as to how these light chains arrange when myosin is bound to actin filaments.

**R: Our results are similar to the results of Robert-Paganin et al., which we have cited in the manuscript, but the actomyosin interface is described at higher resolution. Importantly, also ELC and MTIP are located in the rigor state complex for the first time, albeit at lower resolution. We discuss concisely similarities and differences.**

The models described in this manuscript are compared to isolated structures of either myosins or actin filaments, but no comparison with the published decorated PfAct1-PfMyoA filaments is presented.

**R: We have compared our structures to high-resolution complexes in Figure S7 and identified the core contact interface residues, as identified by Robert-Paganin *et al*. in Figure S7 previously. Now, we directly include values for their structure (pdbid: 7aln) in Fig. S7. At the interface of Act1:MyoA, our higher resolution structure provides more accurate information. In addition, we include the rigor state of the new MyoV complex (pdbid: 7plu), which has recently been published. We describe the main difference in the text to Robert-Paganin *et al.* (2021) as follows:**

*“The MyoA:Act1 complex described by Robert-Paganin et al. (2021) has slightly different helical parameters, half a degree in twist and 1 Å larger rise. Largest differences are seen in HCM loop and at the tip of HLH with overall root mean square deviation ~1.4 Å.”* **(p. 12)**

*“The TEDS phosphorylation site [49], T417, located at the tip of the HCM-loop, is solvent accessible. Interestingly, our structural study of the actomosin complex did not require phosphomimetic mutation T417D in contrast to studies of the truncated MyoA [23], which results in a shift of the position of the HCM loop with respect MyoA and where the aspartic acid contributes buried surface area to the interaction However, the density map does not support its phosphorylation.”* ***(p. 16)***

The authors also claim that they identified a novel pocket in actin filaments that is absent from other known F-actin structures available. However, no in vitro or in vivo experiment support their hypothesis that this pocket could accommodate a solute.

**R: The geometry of the pocket and amino acid residues are well-established by our high-resolution observations. We have identified sequence variation and disease mutations associated residues lining the pocket. We do agree that further in vitro and in vivo experiments will be needed to investigate the importance of many of the findings from this work, but this would be a major project in itself, which we view as beyond the scope of this manuscript.**

Reviewer #3: In this manuscript, Vahokoski and colleagues report a 2.6-A-resolution Cryo-EM structure of Plasmodium falciparum’s actin isoform Act1 stabilized with jasplakinolide. Additionally, they report the structure of Act1 decorated with Plasmodium’s myosin MyoA and two associated light chains at 3.1 A resolution. Parasites from the apicomplexa phylum – like plasmodium – use a specialised type of motility known as gliding, which requires this actomyosin complex as its driving motor. As such, the structures presented in this manuscript are clearly of high interest, as they are a good pharmacological target. The high resolution of both structures, and particularly of the naked actin filaments, represent a large technical advance over the available structures. The research presented in the paper is sound and I would, in general, recommend the paper for publications. However, I found that in its current version, the results – especially some of the figures – could be presented in a much clearer way.

**R: We thank the reviewer for the positive comments and for the suggestions to improve the clarity of the manuscript. We have tried to take all these comments into account, as detailed below.**

**Part II – Major Issues: Key Experiments Required for Acceptance**
Please use this section to detail the key new experiments or modifications of existing experiments that should be absolutely required to validate study conclusions.

Generally, there should be no more than 3 such required experiments or major modifications for a "Major Revision" recommendation. If more than 3 experiments are necessary to validate the study conclusions, then you are encouraged to recommend "Reject".

Reviewer #1: Slightly disappointing that there were no biological experiments to probe the structural observations at the myosin-actin and light chain interfaces. If any updates on this are available or feasible within a reasonable timeframe it would enhance the paper.

**R: We have provided new functional measurements on the MyoA-light chain complex power stoke.**

Reviewer #2: From the low-resolution structure of decorated filaments with MyoA:ELC:MTIP, the authors propose an arrangement within the parasite PM-IMC space suggesting that there is no space to accomodate GAC, which has a Dmax of about 25 nm. Since the authors have previously published a low resolution SAXS GAC model, it would be fair to discuss and model how such a glideosome structure could be formed in such a confined space. The current Fig. 2 should be expanded to also contain GAC.

**R: We now include GAC in Figure 2 to show the relative sizes within the PM-IMC space.**

No in vitro or in vivo experiments were done to support structural findings. The authors claim that there is a specific cavity within PfAct1 filaments that is absent from other known structures but they have not validated experimentally the importance of this pocket. Mutagenesis of residues that line this pocket, for example Val77, and in vitro tests of mutated MyoA function would add credit to the structural findings.

**R: We have now conducted optical trapping experiments with our recombinant MyoA:ELC:MTIP complex, and show that the MyoA complex produces an ATP-dependent power stroke of ~4.5 nm. Given the amount of time required, we view extensive mutagenesis analysis of actin to be outside of the scope of our manuscript. This is naturally something we wish to work on in the future.**

The current structure is compared to the structures of isolated PfMyoA and isolated PfAct1 filaments but no comparison with the latest PfMyoA-PfAct1 complex structure, PDB 7ALN, is done. The latter structure is the closest to the one determined by the authors and it is critical that the authors comment on it.
The authors observed very similar fractional buried surface area of MyoA residues in the actin contacting segments with other MyoA-Actin structures, although sequence divergence is high. They also details the similarities and differences for each of aminoacid (Fig. S7) and especially compare their results with the “conserved and ancillary” interface residues defined by Robert-Paganin et al (2021). It is not clear from the text if the latter differences stem from the analysis method or from strudtural divergence. To help the readers understand better, it would be good to include the other plasmodium MyoA structure (PDB: 7aln) in the table of Fig. S7.

**R: We now directly include their structure (pdbid: 7ALN) and, as noted above, have identified a difference in the HCM loop.**

Reviewer #3: Here are some suggestions on how the manuscript could be improved, and some comments that I think need clarification.

- Some of the figures are rather hard to follow. For instance, it is really difficult to dissect the interaction between MyoA and Act1 in Fig3, as the assignment of MyoA’s regions is somehow ambiguous. Colour coding like in Fig S6 would make the figure much easier to understand.

**R: We have done this.**

It is also not at all clear to me what’s the extent of the cavity shown in Fig4, nor what is the role of all the amino acids highlighted in the panels. Moreover, while panels A and B are the same structure, panel B is rotated so it’s hard to relate both snapshots. I find that the cavity appears much clearer in Movie S4. Would a similar representation be possible in the figure?

**R: We now keep the common views in Figure 4. We now include panel B in a separate Figure 5 along with a diagram (ligplot) of ligand interactions.**

FigS5 does not bring much. Except for the single actin monomer, it is impossible to see whether or not the models fit the density. Movie S2 is much better at that. FigS8 feels unnecessary since it is basically Fig4B but with less information.

**R: We have removed Figure S8 but have retained Figure S5.**

- The resolution estimation is somehow inconsistent throughout the manuscript. For instance, the methods state for actomyosin that (l.360-362) “The estimated global resolution reached 3.1 Å calculated with Blocres in Bsoft package version 1.8.6 with a Fourier shell correlation threshold of 0.3)”. In FigS1 however it is clear that this was done with Relion, as for Act1. Could this be just a mix up with the local resolution estimation?

**R: Yes, we meant to say global resolution and have corrected this.**

The local resolution estimation is confusing as well. For example, the resolution scale in FigS4 goes between 2.1 and 2.5, all values below the average resolution of 2.6 (threshold 0.143). How is this possible? Fig S3 shows the local resolution for actomyosin. In those, the resolution was estimated with a different threshold (0.3). What’s the reasoning behind this?

**R: We now consistently use a resolution threshold of 0.3 for local resolution estimation because it is more conservative and preferable because of overestimates associated with calculating local resolution using a small box size (See discussion Cardone *et al.* 2013:** [**https://doi.org/10.1016/j.jsb.2013.08.002**](https://doi.org/10.1016/j.jsb.2013.08.002)**). This has fixed the problem the reviewer recognised in Figure S4.**

- A more detailed description of the Act1/MyoA interaction missing. For instance, Fig3 provides the residues from MyoA involved in the interaction, but Act1 is simply shown as surface. This might be important, considering that the residues in these interfaces aren’t strongly evolutionary conserved (as shown in the text), and Act1 is relatively divergent from other actins as well.

**R: Figure 3 now includes a transparent surface for actin in panels b, c, and d.**

Similarly, in page 14 the manuscripts states “...forms a second important interface to Act1 [...] that is complementary in shape and hydrophobic in nature. Surprisingly, there are no side chain interactions contributing to this interface, which is rather mediated by main chain atoms”. How can an interaction mediated by H-bonds be hydrophobic?

**R: We apologize for the confusion, and we have reformulated the text as follows:**

*“The HCM loop* ***(Fig. 3B)*** *forms a second important interface to Act1 (buried surface area 284 Å^2^) that is complementary in shape, and we detect no hydrogen bonds at the interface.”*

- X-ray structures of MyoA alone or in complex with its light chains are already available. As with other myosins, there’s small conformational changes upon binding to F-actin. Although these changes are already described in the text (l 204-209), it would be easier to illustrate this with a structural alignment. In addition, there is another MyoA (without light chains) and Act1 structure already available (ref 23). I understand that the preprint for this manuscript precedes Dorit Hanein’s paper (ref 23), but I think a comparison between the two structures would be interesting, and possibly shed some light into the effect of the light chains on myosin.

**R: We have modified Figure S7 to include the structure for comparison, and we have high-lighted the major difference within the main text.**

Likewise, in line 170 the manuscript states that “ELC and MTIP bind to the MyoA neck in a compact manner, presumably stiffening the neck region so it acts as a relatively rigid lever arm…”. Isn’t this the case in all myosins? A figure comparing some of the known structures would help strengthening the point.

**R: To address this point, we have now performed optical trapping assays to estimate the MyoA power stroke, as presented in Figure 2. The MyoA-light chain complex power stroke is similar to other myosins with two light chains. We also mention these data in the concluding remarks (p. 16).**

- The way in which Plasmodium actin breaks ATP and releases Pi seems to be very different than what has been observed in other actin homologues. Having the highest resolution structure of F-actin published to date, it feels as a missed opportunity not to speculate about the structural basis for these differences. Is the configuration of the active site different from other actin filaments? Could the cavity seen in the structure explain the differences in Pi egress kinetics?

**R: We have modified the text as follow (lines 265-278):**
*“The end of the cavity is formed by the side chains of Asn116 and Trp80* ***(Fig. 4, Movie S4)****. In monomeric Act1, the cavity is much shorter compared to the F-form* ***(Fig. 4C).*** *In muscle F-actin, the cavity is limited by the bulkier Ile76 instead of Val77 in Act1* ***(Fig. 4).*** *Notably, residue 76/77 is an Ile in α- and γ-actins, but is also Val in β-actin as well as plant and yeast actins, which release phosphate faster than muscle actin [48, 49]. We have previously noted the importance of Gly115 substitution to alanine in Act1; alanine at this position slows down phosphate release compared to glycine, in particular in the Mg-bound form [15, 28]. In skeletal or smooth muscle α-actin, Asn115 and Arg116 are sites of disease-associated mutations (N115S, N115T, R116Q, R116H) [50, 51]. In yeast, mutations N115T and R116Q affect both nucleotide exchange as well as polymerization kinetics, in particular in the nucleation phase [52]. Therefore, these features of Act1 active site may be relevant to understand actins generally.”*

- It is not clear to me whether Ser19 is phosphorylated in the structure in this manuscript. Is there independent evidence showing that it is? Was this expected from protein production? The extra density shown in Fig 1 does not look completely convincing. Perhaps doing FDR analysis (with the SPOC package from Carsten Sachse’s group) would help determining whether this is a real feature of the map. If this was indeed a phosphorylation in the map, could the atomic model provide an explanation of how this PTM would affect the function of myosin?

**R: We have improved the panel on Figure 1. We have confirmed by mass spectrometry that the side-chain is ~50% phosphorylated.**

*“The phosphorylation of Ser19 in the MyoA:MTIP:ELC complex is visible in the density map (Fig. 1) and was confirmed using mass spectrometry, showing ~50% phosphorylation of the relevant peptide.”* **(p. 12, lines 240-243)**

On a related note, the manuscript mentions (p. 15) that T417 is not phosphorylated, and contrary to ref 23, a phosphomimetic mutation wasn’t required for proper actin decoration. Any idea why this would be the case?

**R: We now say in the text that the HCM loop containing T417D is different and facilitates a slightly different interaction. We are not sure why reference 23 required a mutant. Comparing our images with those in reference 23, we have much more complete decoration, which may have led to our having a higher resolution structure.**

**Part III – Minor Issues: Editorial and Data Presentation Modifications**
Please use this section for editorial suggestions as well as relatively minor modifications of existing data that would enhance clarity.

Reviewer #1: (No Response)

Reviewer #2: Lines 211-213: The position of the apicomplexan-specific N-terminal helix links the SH3 and motor domain and contains Ser19. With their structure, the authors are now in a better position to extrapolate how Ser19 phosphorylation status will influence MyoA motor function.

**R: Unfortunately, we would need MyoA in multiple nucleotide states in order to understand this better, and the structure at present does not inform about its function.**

The positioning of Ser15 side chain contradicts the previous findings of its importance for coordinating ADP beta-phosphate. Although this is mentioned in the text, the authors do not comment on the possible explanations of why they observe this difference.

**R: We think our differences are due to having a high-resolution filament structure by cryo-EM, whereas the previous view has come from crystallography of monomeric actin. For clarification, we have modified the text as below.**

*“Based on crystal structures of monomeric actin, the side chain of Ser15 (14 in canonical actins) has been established as a nucleotide sensor, which is turned away from bound ATP but hydrogen bonded to the β-phosphate of ADP [53]. In our structure of the ADP-bound filamentous Act1, the side chain of Ser15 points away from the nucleotide, not contacting the ADP phosphates, thus more closely resembling the conformation in crystal structures of the ATP-bound state* ***(Fig. 5)****. Instead, the Ser15 main chain NH group interacts with the β-phosphate.”* ***(p. 13-14 )***

Line 268-269: Sentence starting with :” Within loop 3, Myo 1b has...” needs rephrasing as it is not clear what contacts are described here.

**R: This now reads:** *"Within loop 3, Myo 1b has extensive contacts with actin that are not present in other myosins."***.**

Description in atomic details of the HTH-actin interface would greatly benefit from showing a zoomed-in view of the amino acid involved.

**R: We now have an improved Figure 3c.**

Reviewer #3: - Line 290 says “Human Cardio-Myopathy” instead of hypertrophic cardiomyopathy.

**R: Fixed.**

- In page 16 the text says “samples were prepared on Quantifoil 2/2, 200 mesh air- glowed grids”. The word discharged is missing.

**R: Fixed.**
